# Silver–Organic Complex in Photosensitive Silver Pastes for Enhanced Resolution and Aspect Ratio

**DOI:** 10.1021/acs.langmuir.4c02158

**Published:** 2024-08-13

**Authors:** Jyun-Hao Chen, Yen-Ting Liu, Chia-Chun Hsieh, Yi-Cheng Chou, Chun-Hu Chen

**Affiliations:** Department of Chemistry, National Sun Yat-sen University, Kaohsiung, Taiwan 80424

## Abstract

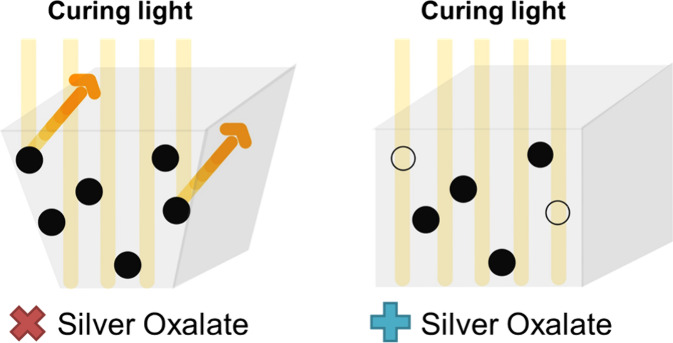

Traditional screen printing is an easy approach commonly used for conductive pattern fabrication of electronics but lacks high resolution. Photolithography offers better resolution but is complex. Photosensitive silver pastes (PSP) combine the benefits of both but suffer from undercut issues, causing uneven etching, decreased interfacial adhesion, and thus poor resolutions. In this study, we explore the use of molecular precursors (i.e., silver oxalate) to replace metallic silver particles and enhance the depth of light penetration. Our findings demonstrate a successful solution to the undercut issue, achieving an undercut index of 1.0, indicating an undercut-free scenario and enabling higher resolutions in line and pattern formation. Additionally, our research confirms the feasibility of multilayer stacking of photosensitive pastes, achieving unprecedented aspect ratios in line patterns. By replacing 25% of micrometer silver powder with silver oxalate (PSP-25), we achieved optimal line widths as fine as 10 μm. The three-layer stack of PSP-25 reached a substantial aspect ratio with a height of 29.4 μm and an optimal fringe pattern resolution of 10 μm line width with a 15 μm aisle width. Utilization of silver oxalate was observed to slightly expand the line width, likely due to light scattering by the fine silver nanoparticles (∼40 nm) formed during the photodecomposition of silver oxalate.

## Introduction

In modern electronics, the demand for compact integration and miniaturization of circuit patterns is crucial.^1−3^ Low-temperature co-fired ceramic (LTCC) has emerged as a popular method to achieve high-resolution component miniaturization due to its low material loss and high-level integration capabilities.^4−7^ Traditionally, conductive patterns in LTCC are created using screen printing, a process where metallic powders mixed with polymers are applied through pattern masks onto substrates.^8−10^ After patterns were transferred, thermal treatment was conducted to eliminate the organic residue, resulting in a direct transfer of the patterns into metallic ones (e.g., Ag). Although widely used, screen printing struggles with achieving resolutions finer than 100 μm, despite advanced techniques that push this to around 40–50 μm.^11−15^ On the other hand, photolithography-based technologies are capable of realizing much finer resolutions.^16−18^ Unlike straightforward screen printing, photolithography involves complex steps such as etching, metal deposition, lift-off, etc., to generate metal wires and patterns on the substrates. Therefore, integrating the simplicity of screen printing with the high resolution of photolithography is highly desirable. This has led to the development of photosensitive pastes (PSP) also known as photoimageable pastes,^19−24^ which combine metallic silver powders and photoresists to enable easier pattern transfer through light exposure and development process.^25,26^

Photosensitive pastes typically contain about 65–70% silver micrometer powder and 30–35% organic-based photoresist.^21,22^ When exposed to light, these pastes not only cure but also encapsulate the silver particles, allowing the unexposed areas to be washed away, followed by thermal annealing to achieve high-resolution patterns.^27^ These photolithography-like characteristics enable a high resolution of transferred patterns in the resultants. The entire procedure is simple and capable of solving the low-resolution drawback of screen printing. Kim et al. demonstrated that by altering the molecular weight of polymeric binder, line resolution can be narrowed down to 20 μm.^28^ Another study presented a sub-20 μm silver line using PSP by adopting a special back-side irradiation approach.^29^

Despite their advantages, PSPs suffer from a major issue of undercut—where the uneven penetration of light causes incomplete curing at the bottom, leading to weak adhesion and easy peel-off during development (also see Scheme 1).^28,29^ The occurrence of undercut decreases the adhesion strength of the lower edge of patterns/lines and thus easily peels off during development, which is more severe for those with finer line width. Moreover, the thickness of the pastes cannot exceed the depth penetrated by light, which restricts the potential of photosensitive pastes to achieve a higher line resolution.

**Figure 1:**
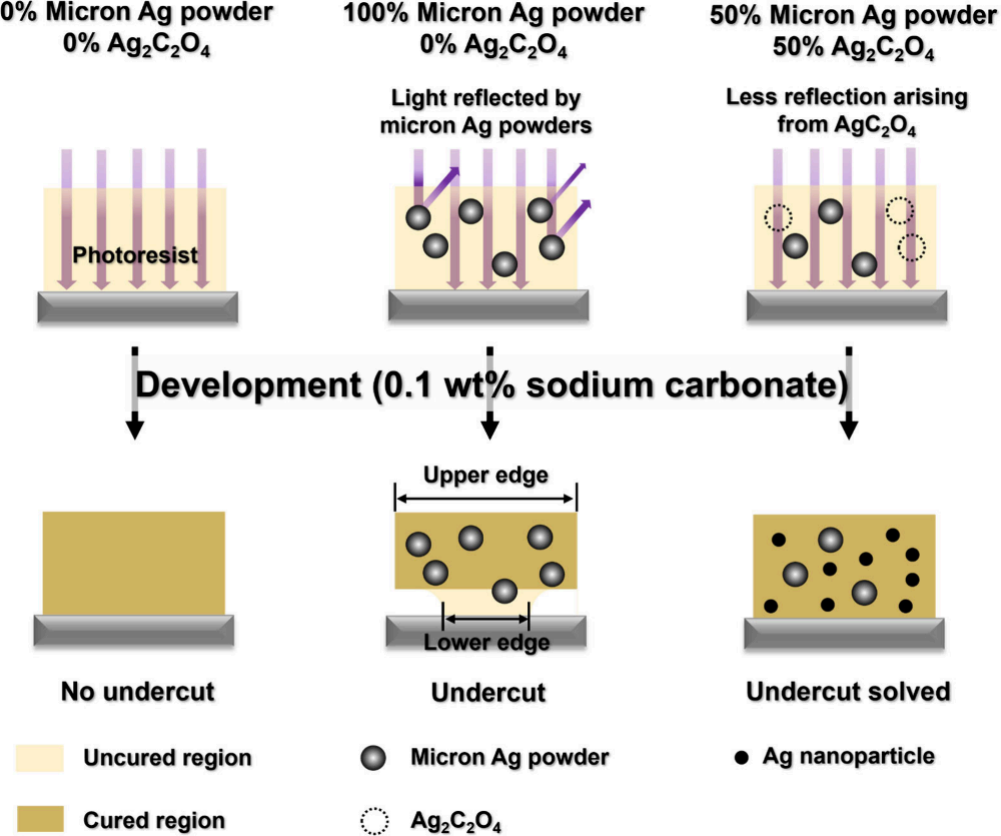
Mechanism of Undercut Formationa

a In the left pathway, no micron silver particles are present, allowing the incident light to penetrate fully to the bottom of the paste (i.e., the photoresist), where the lower edge is completely cured. This fully cured area resists the etching solution during development, preventing undercut formation. In the middle pathway, the presence of micron silver particles in the conventional PSP obstructs the light, leaving the bottom area uncured. As a result, undercut occurs after development, and the lower edge is shorter than the upper edge. In the right pathway, the incorporation of silver organic complexes in place of micron silver particles mitigates the blocking of incident light, ensuring sufficient light reaches the bottom of the paste. This adjustment effectively addresses the undercut issue. The presence of micron silver powder in conventional setups tends to reflect the incident light, leading to inadequate light penetration from the top to the bottom of the paste, resulting in undercut after development.

Given that the undercut phenomena primarily arise from the facile reflection of incident light by metallic silver particles,^28^ we propose to use molecular precursors of silver, rather than the metallic particle format, to enhance the light penetration depth in the PSP (see Scheme 1). The interaction between molecular complex and incident light is more featured on light absorption^30−33^ (i.e., between the gap of the highest occupied molecular orbital (HOMO) and the lowest unoccupied molecular orbital (LUMO)), rather than the reflection pathway. By replacing metallic silver particles with a molecular complex, the light reflection effect should be inhibited in PSP. Of course, the wavelength of the incident light should be selected to avoid the region of strong absorption (i.e., within the HOMO/LUMO gap) of the complex. This will allow light to still penetrate down to the bottom of the paste, thereby eliminating the undercut issue. In addition, we also need the molecular precursors to be easily converted to metallic silver. Thus, we adopted photosensitive silver precursors, which possess a rapid redox reaction pair that can be easily initiated at room temperature with light irradiation, enabling higher degrees of conversion from the molecular form into silver particle form and thus gaining a higher conductivity. Therefore, we selected silver oxalate as the silver organic complex to investigate in this work, which can be both thermally and photochemically decomposed into metallic silver.^30^

In this study, we synthesized silver oxalate and used it to replace various amounts of commercial micrometer-sized silver particles in photosensitive paste (PSP) samples. The findings demonstrate that silver oxalate effectively addresses the undercut issue, enabling the achievement of higher resolutions in line and pattern formation. Building on this success, we explored a multilayer PSP stack designed to achieve high aspect ratios in line patterns, achieving a height difference of up to 30 μm—an outcome not previously realized with PSP technologies. By addressing the undercut issue, multilayer PSP stack can be realized to fulfill the evolving demands of future electronic devices.

## Experimental Section

### Synthesis of Silver Oxalate

Silver oxalate was synthesized by mixing a 1 M sodium oxalate (NaC_2_O_4_) and 1 M silver nitrate (AgNO_3_) solution at room temperature. The as-obtained precipitate was washed with DI water, ethanol, and acetone twice and then dried using a vacuum dryer for 24 h.

### Preparation of Photosensitive Silver Paste

The photosensitive silver paste was prepared by mixing a specific ratio of bis(acyl)phosphine oxide photoinitiators (0.2%), benzophenone derivatives (0.8%) as the photosensitizer, acrylic monomers (6.7%), polyether dispersants (0.8%), methoxy propyl acetate, diethylene glycol hexyl ether (solvent) (7.5%), and the micron silver powder (Ample Electronic Technology Company; no purification or grinding step was conducted before use) together and homogenized by a triple roller mill at room temperature.^13,22,34^ The ratio of the total content of organic chemicals to the micrometer silver powder was kept at 3:7. To study the role of silver oxalate in solving undercut issues, several weight percent of the micron silver powder (0%, 13%, 25%, and 50%) used in the original pastes was substituted by silver oxalate, and the resultant samples are named PSP-0 (the same as the pristine samples without adding any silver oxalate), PSP-13, PSP-25, and PSP-50, respectively.

### Fabrication of a Silver Line Pattern

The as-made PSP samples were used following a screen-print procedure on a 6 × 5 cm^2^ Al_2_O_3_ substrate. A screen-printing frame was placed first on top of the Al_2_O_3_ substrate with a 1.8 mm spacer, and the printing of PSP samples proceeded using a commercial screen printer (MT-320TV, Microtec). The printed PSP samples were then dried at 75 °C for 15 min to eliminate the solvents. The exposure process (Group Up Industrial Co., Ltd.) was carried out under light irradiation (405 nm wavelength) for 1 min with a photomask covering on top of the sprayed PSP. The development of patterns was carried out using a developer of 0.1 wt % sodium carbonate for 1 min. There are two different calcination procedures: the first one was done within a furnace (Thermo Scientific, Thermolyne) at 850 °C in the air for 2 h; the second one was to hold the calcination temperature at 200 °C for 2 h and then 850 °C for another 2 h. The multilayer printing procedure (i.e., two-layer and three-layer) was carried out using the same step but printed two times and three times of the PSP before soft baking.

### Material Characterization

The cross-sectional image and undercut index of the silver circuit were acquired by an FEI Inspect F50 field-emission scanning electron microscopy (FESEM) instrument with 10 kV accelerated voltage. The XRD patterns were obtained by a Bruker D2 Phaser diffractometer with Cu Kα X-ray (λ = 1.5418 Å) radiation. The UV–vis transmittance (%) results were collected using a JASCO V-630 double-beam spectrophotometer.^35^ The doctor blade method was used to prepare the samples for the transmittance measurement by controlling the depositing area (2.5 × 1 cm^2^) and the paste loading (11.3 ± 3.5 mg) of the PSP samples. The resolutions of the silver lines were examined using a Keyence VHX-900F 3D optical microscope and are defined by the line width and interline spacing. The thermogravimetric analysis (TGA) was conducted in the temperature range of 30–900 °C using a PerkinElmer TGA 4000 instrument with a heating rate of 10 °C/min under ambient air conditions. The resistance of silver circuits was measured by using a Keithley 2400 source meter. The bulk resistivity can be determined by the following equation:^36,37^
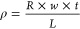
1where ρ is the bulk resistivity (Ω·cm), *R* is the resistance (Ω), *w* is the line width (cm), *t* is the thickness (cm), and *L* is the line length (cm).

## Results and Discussion

### Characterization and Photoactivity of Silver Oxalate

The XRD patterns of the as-synthesized silver oxalate with different exposure times and the reference patterns are shown in Figure 1a. The XRD results of the as-synthesized products are very consistent with the reference pattern of silver oxalate (JCPDS-22-1335) without any appreciable impurity. To assess the photodecomposition of silver oxalate under light irradiation (405 nm), the products post-2 h exposure were analyzed using XRD, revealing additional peaks indicative of metallic silver (JCPDS 87-0717). Extending the irradiation from 2 to 4 h increased the relative peak intensities for metallic silver, confirming its formation from silver oxalate under light exposure.

**Figure 1:**
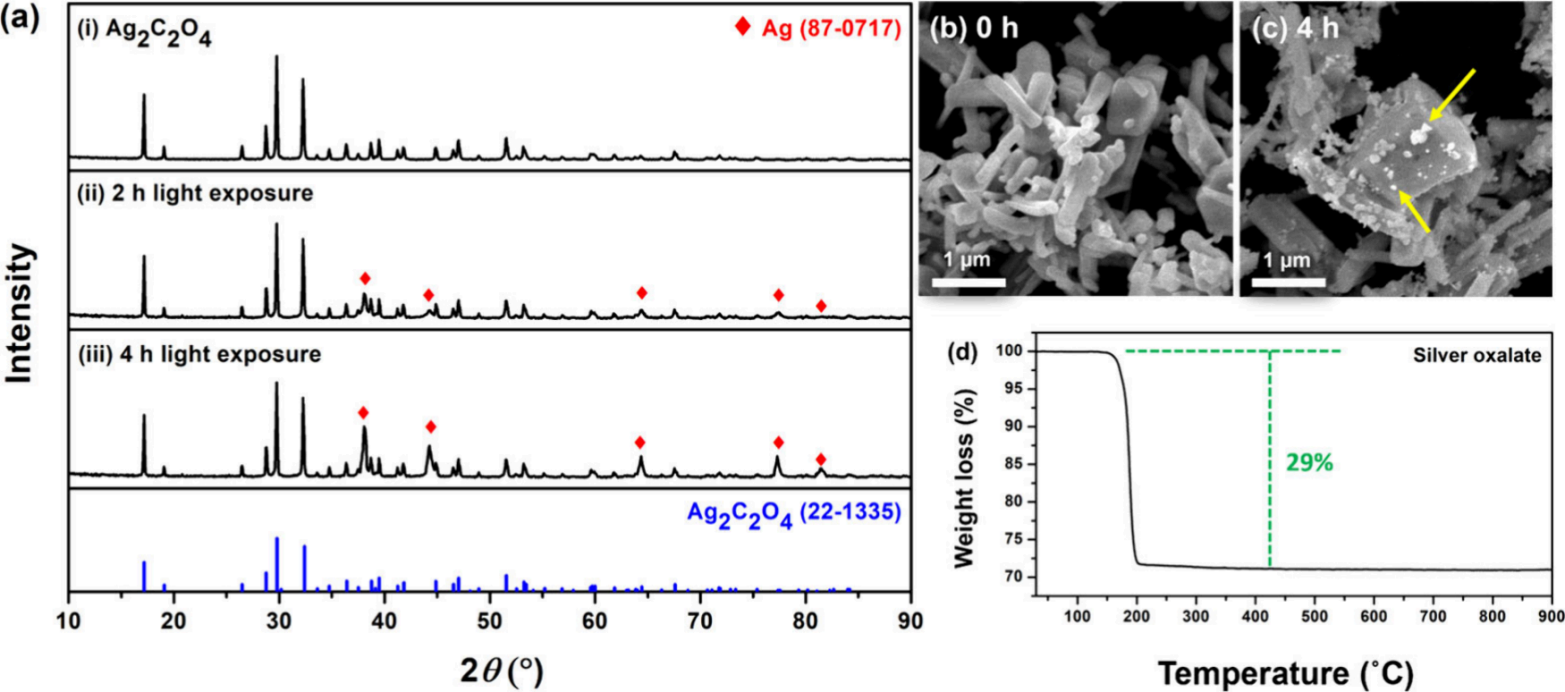
Structural characterization and photoactivity of silver oxalate. (a) XRD patterns of the as-synthesized silver oxalate (i) and silver oxalate after light exposure for 2 h (ii) and 4 h (iii). The reference patterns for metallic silver (JCPDS No. 87-0717, red diamonds) and silver oxalate (JCPDS 22-1335, blue pattern) are included for comparison. (b) SEM images of the as-synthesized silver oxalate showing elongated particles with smooth surfaces. (c) SEM images of silver oxalate after 4 h exposure, showing growth of fine metallic silver particles (200–300 nm in diameter) on the surface, indicated by yellow arrows. (d) TGA graph of the as-synthesized silver oxalate under a nitrogen atmosphere, showing a weight loss of 28% at 140–200 °C, consistent with the theoretical oxalate ion content of 29%.

The SEM images (Figure 1b) revealed that the as-synthesized silver oxalate shows an elongated shape with a smooth surface with a particle size distribution of 1.173 ± 0.325 μm at the long axis and 0.348 ± 0.099 μm at the short axis (Figure S2). After 4 h of irradiation, fine particles (200–300 nm in diameter) are observed (Figure 1c), corresponding to the metallic silver peaks observed in XRD results. All of the results above confirm the photodecomposition of silver oxalate under 405 nm irradiation, as shown in eq 2:
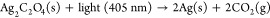
2The results of thermal stability of the as-obtained silver oxalate evaluated by TGA show a weight loss of 28% at 140–200 °C, which is in high agreement with the theoretical oxalate ion contents (29%) in silver oxalate.

### Undercut of the PSP Samples

**Figure 2:**
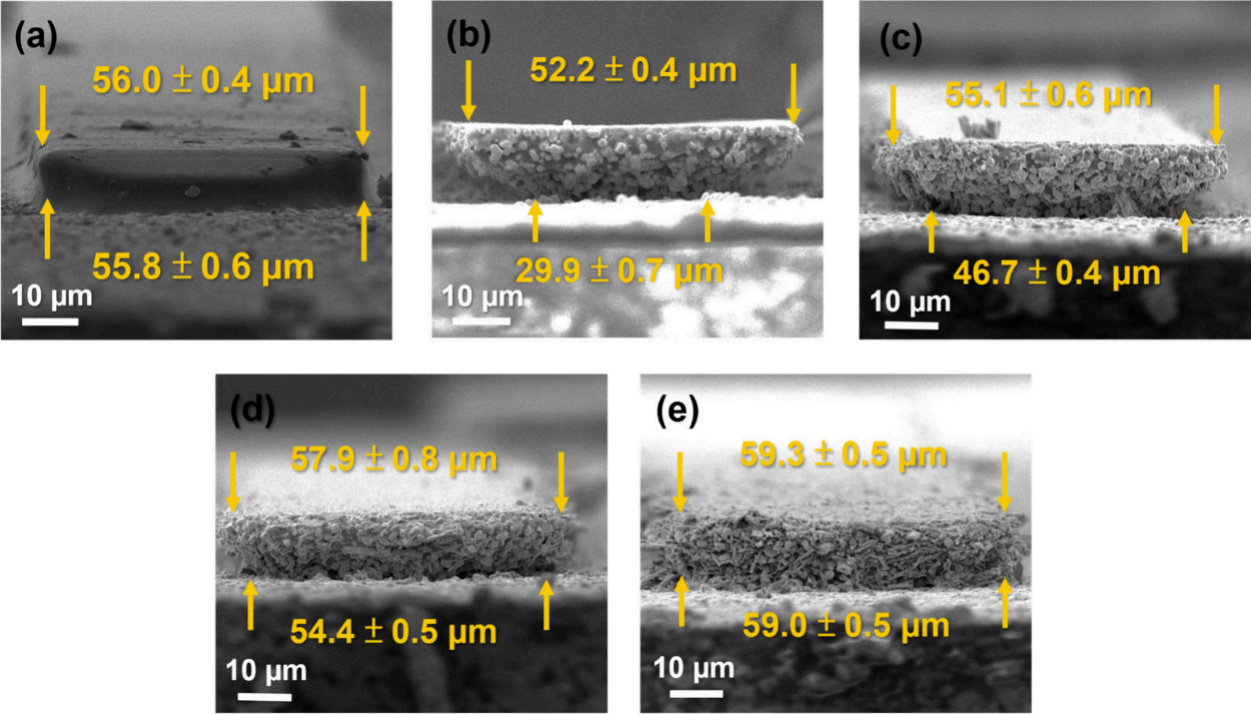
SEM images of cross sections of the PSP samples to reveal the undercut issue. (a) Blank sample (without silver particles and silver oxalate). (b) PSP-0, with severe undercut. (c) PSP-13, showing the reduced degrees of undercut. (d) PSP-25, with the further reduced undercut issue. (e) PSP-50, showing no undercut to be observed. The corresponding undercut indices are 1.0, 1.7, 1.2, 1.1, and 1.0, respectively.

The cross-section SEM images of the developed silver lines were used to evaluate the degrees of the undercut (Figure 2). For quantitatively indexing undercut phenomena, we introduced an index of undercut defined by the following equation:
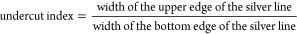
3According to eq 3, an undercut-free scenario would result in the index value to be one. The greater the deviation from one, the more severe the undercut issue becomes. By comparing the undercut indices, we can determine the effectiveness of our proposed silver–organic approach in addressing the undercut issues. For the silver oxalate-free PSP-0 samples, which still contain micrometer-sized silver particles, the undercut index is 1.74 (with the top edge width of 52.2 ± 0.4 μm and the bottom width of 29.9 ± 0.7 μm). Such severe undercut is commonly observed in the literature for the silver particle-based PSP.^28,29,38^ To investigate the effect of micrometer silver particles on the undercut issue, a blank sample (PSP-0 without adding any micrometer silver particles) was tested under the same conditions, yielding an undercut index of one (Figure 2a). This clearly indicates that the addition of the micron silver particles is the direct cause of undercut.

Conceptually, the absence of undercut (i.e., the undercut index = 1) can be achieved only when the irradiation completely cures the exposed area vertically (from the bottom to the top edge). These results further confirm that light cannot completely penetrate the entire thickness of the PSP in the presence of micrometer-sized silver particles. The difficulty of light penetration should be due to reflection and/or scattering. The 405 nm wavelength of light may not be effectively scattered by the micron-sized particles, since there is a size mismatch so any significant light scattering cannot be reasoned.^39−44^ Therefore, reflection caused by the micrometer silver particles should be a more reasonable cause of poor light penetration.

We further measured the UV–vis transmittance on the PSP samples. As shown in Figure S3, the increasing trend of the transmittance (%) at 405 nm is observed as the following: PSP-0 (0.56%) < PSP-13 (0.83%) < PSP-25 (2.03%) < PSP-50 (2.94%). These results further support that the higher contents of silver oxalate enhance the light penetration depth.

By using silver oxalate as the replacement for the micrometer silver particles, the undercut index for each sample is 1.2 ± 0.2 (the top edge width of 55.1 ± 0.6 μm, and the bottom width of 46.7 ± 0.4 μm) for PSP-13 (Figure 2c); 1.1 ± 0.1 (the top edge width of 57.9 ± 0.8 μm, and the bottom width of 54.4 ± 0.5 μm) for PSP-25 (Figure 2d); and 1.0 ± 0.1 (the top edge width of 59.3 ± 0.5 μm, and the bottom width of 59.0 ± 0.5 μm)) for PSP-50 (Figure 2e). These results indicate that substituting silver oxalate is highly effective in inhibiting the undercut issue. Lowering the contents of micron silver particles via photosensitive silver–organic complex inhibits the reflection, allowing the light to completely penetrate through the entire paste. The in situ conversion of silver oxalate into silver particles under the 1 min exposure is anticipated to yield much smaller particle sizes as compared to those formed under a 4 h irradiation (0.2–0.3 μm, see Figure 1c). With such ultrafine particle sizes, significant reflection is minimized. Hence, the incident light can readily penetrate these silver oxalate-added pastes, curing the bottom parts to solve the undercut issue.

### Resolution Evaluation of the PSP Samples

We also studied the impact of the photosensitive silver–organic complex on the resolution of the developed silver line, focusing on two types of resolutions: single-line resolution and interline resolution. The single-line resolution represents the finest line width that can remain stably immobilized on the substrates under the undercut effect. When undercut happens, the bottom parts are usually overetched within the optimal developing time. Therefore, when the line width is approaching narrower values, the probability of pattern detachment would significantly increase.

**Figure 3:**
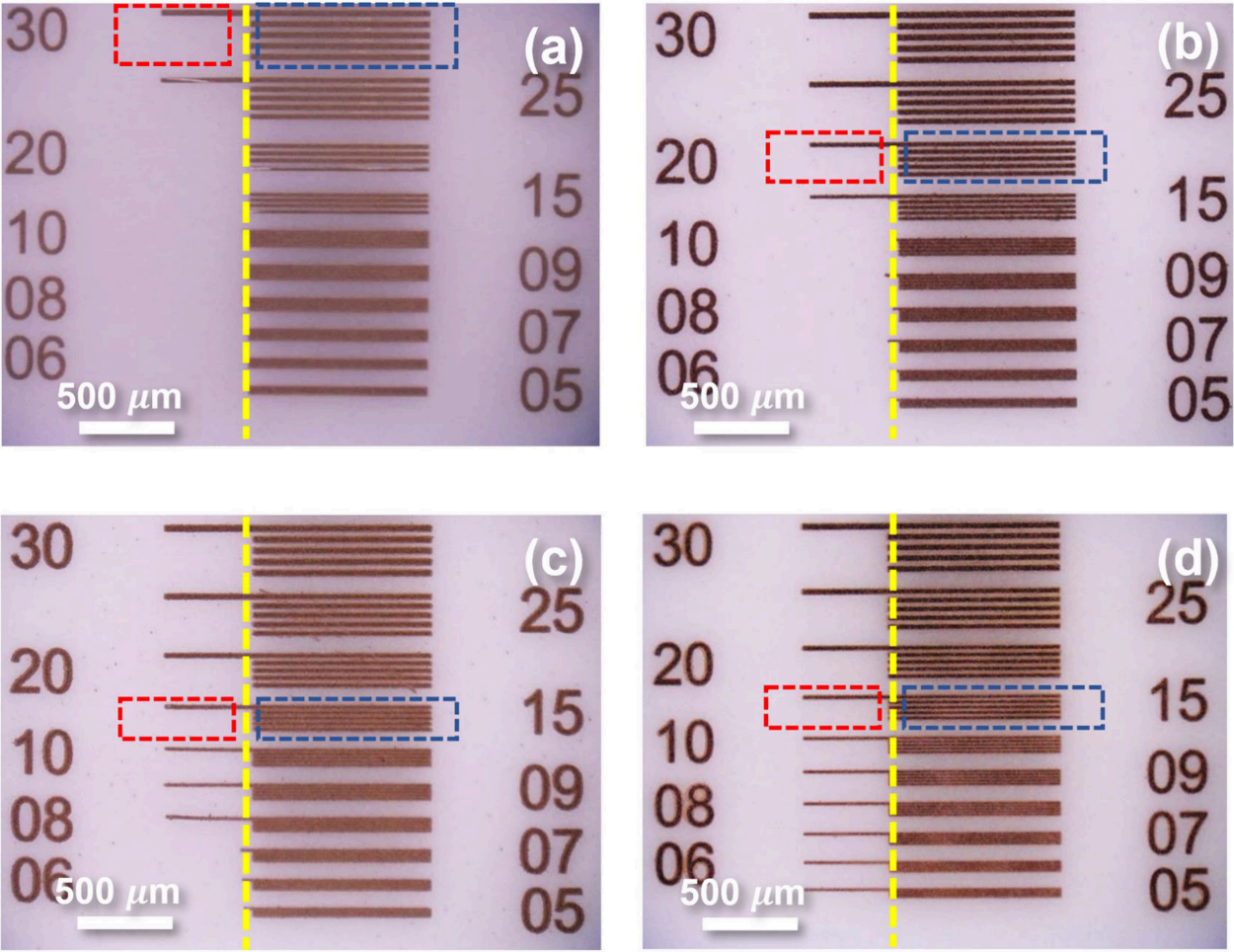
Developed patterns of the PSP samples. The single-line area is on the left side of the yellow dashed line, showing the finest robust width against undercut issues. The fringe pattern area on the right demonstrates the optimal interline (line/aisle) resolution. (a) PCP-0: the optimal single-line resolution of 30 μm (red-dashed box), because of the partial peel-off at the 25 μm row; the optimal fringe pattern resolution of 30 μm row. (b) PSP-13: optimal single-line resolution of 15 μm. (c) PSP-25: optimal single-line resolution of 8 μm. (d) PSP-50: optimal single-line resolution of 5 μm.

Figure 3 shows the optical microscope photographs of the developed PSP samples, where the numbers indicate the photomask resolution of the single line area (on the left of the yellow dashed line) and the interval resolutions in the fringe pattern area (on the right of the yellow dashed line). Each number signifies both the single line width and also the line/aisle width of the fringe pattern in the specific row. For example, within the 30 μm row, all of the line width and the gap between each line are designed to be 30 μm on the photomask. The optimal resolution of line width was identified as the finest line width remaining immobilized on the substrate after development, while that of the interval patterns was recognized as the narrowest interval, where each line is still completely free of contact with any other lines. Note that after the development the actual line width of the products may vary from the indicated numbers of the row. Such a design of experiments can allow us to evaluate both what the optimal line width is and the degrees of possible line width expansion in just one test/specimen. We will report both the photomask-defined line widths (as the entire row) and the actual observed line widths of the experiment as a result in the following paragraph.

Based on the single-line area shown in Figure 3a, PSP-0 has the finest line resolution at the 30 μm row, as the 25 μm row already displays partial peel-off. The finest line resolutions for PSP-13 (Figure 3b), PSP-25 (Figure 3c), and PSP-50 (Figure 3d) are at the 15, 8, and 5 μm rows, respectively. Overall, the finest actual line width is determined to be 10 μm in PSP-25. These results show that the single-line resolution can be effectively improved by solving the undercut phenomena. The adhesion of the lower edge can be greatly improved by having a wider contact area with the substrate compared to the highly undercut cases, thereby leading to higher success rates of preserving finer lines in the defined pattern for an improved resolution of PSP.

The optimal interval resolution of the fringe patterns for these PSP samples is as follows: for PSP-0, it is at the 30 μm row with the exact line/aisle width corresponding to 31.0 μm/25 μm; for PSP-13, it is at the 20 μm row with the actual line/aisle width of 18.2 μm/20 μm; and for both PSP-25 and PSP-50, the optimal resolution is at the 15 μm row, where PSP-25 shows the line/aisle width of 10.7 μm/15 μm and PSP-50 shows the line/aisle width of 17.3 μm/15 μm. Although small variations of uneven line/aisle width can be observed, the optimal interval resolution can also be improved by solving the undercut issue. With the improved lower edge adhesion, the interval pattern becomes more robust for a longer time of development that is needed for a more complete removal of narrower aisle areas, where the diffusion kinetics are more difficult than in wider ones. The uneven line/aisle values reveal the occurrence of line width expansion by adding silver oxalate, which is discussed below.

### Line Width Expansion

**Figure 4:**
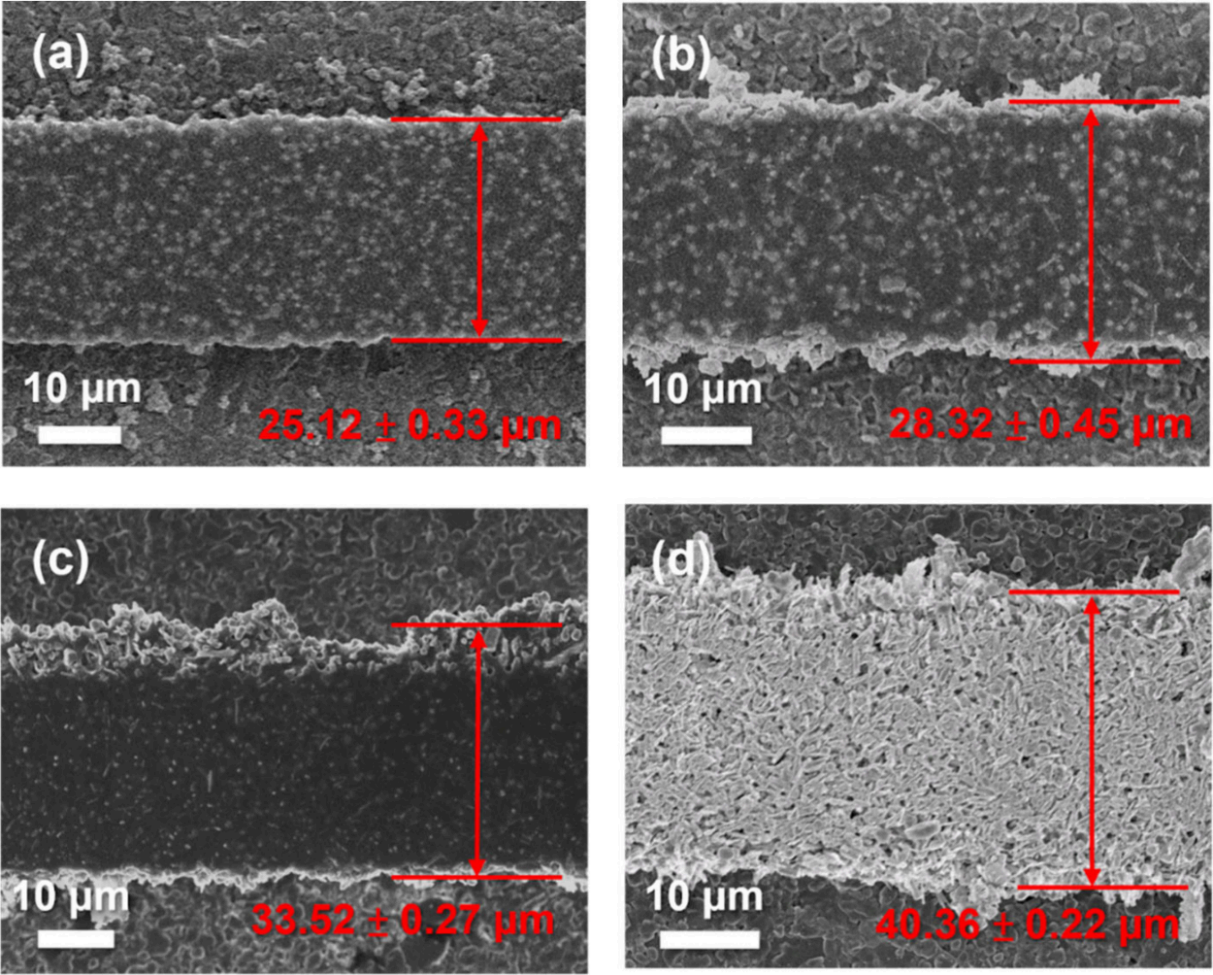
SEM top-view images of the developed silver lines with different silver oxalate contents at the photomask of the 25 μm line. (a) PSP-0: line width of 25.12 ± 0.33 μm, matching the defined photomask. (b) PSP-13: line width of 28.32 ± 0.45 μm, showing slight expansion. (c) PSP-25: line width of 33.52 ± 0.27 μm, showing moderate expansion. (d) PSP-50: line width of 40.36 ± 0.22 μm, showing significant expansion. The line width increases with higher silver oxalate contents.

We systematically evaluated the line width expansion of the silver oxalate-added PSP under the same photomask by conducting a 25 μm line width exposure. The SEM images show that PSP-0 yields a line width of 25.12 ± 0.33 μm, which is well-matched to the photomask definition. By increasing the contents of silver oxalate, the line width is 28.32 ± 0.45 μm for PSP-13 (Figure 4b), 33.52 ± 0.27 μm for PSP-25 (Figure 4c), and 40.36 ± 0.22 μm for PSP-50 (Figure 4d).

Since the silver oxalate does not absorb the incident light, the formation of the ultrafine nanosized silver particles may initiate a light scattering mechanism, leading to an extra curing along the lateral direction and thus causing the line width expansion. Given the ultrafine sizes (∼40 nm and less) of the photoinduced silver nanoparticles on the silver oxalate surface, the Rayleigh scattering process, which occurs when particle sizes are about one-tenth of the incident light wavelength, is likely involved.^37^ Certain parts of such isotropic scattering may lead to curing the lateral parts to different degrees, exceeding the line width defined by the photomask and causing line expansion. The more silver oxalate added, the more photoinduced silver nanoparticles are generated, resulting in more server line width expansion.

### Multilayer Stack of PSP

With the success in solving the undercut issue, one of the classical difficulties in the field–fabrication of high aspect ratio lines via multilayer stacking of PSP—can be addressed.^45,46^ High aspect ratio silver lines are capable of inhibiting resistivity increases when the line width decreases in order to achieve higher resolutions. Multilayer stacking of PSP has been proposed to achieve this goal. Yet, this approach has been challenging for a long time due to the unresolved undercut issue in the past.

**Figure 5:**
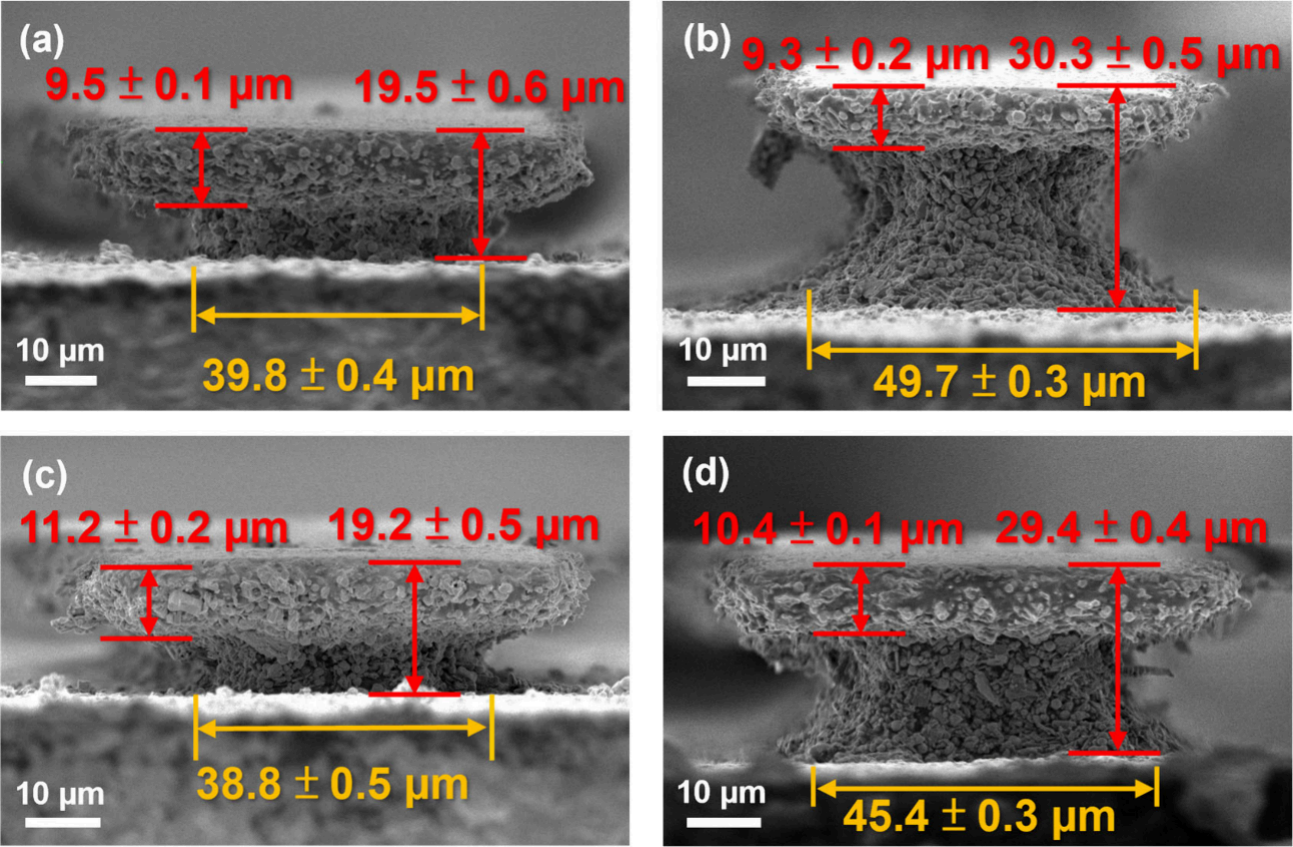
Cross-sectional SEM images of developed multilayer stacked PSP samples. (a) Two-layer stack of PSP-13, showing a height of 19.5 ± 0.6 μm. (b) Three-layer stack of PSP-13, showing a height of 30.3 ± 0.5 μm. (c) Two-layer stack of PSP-25, showing a height of 19.2 ± 0.5 μm. (d) Three-layer stack of PSP-25, showing a height of 29.4 ± 0.4 μm. The images illustrate the successful stacking of multiple PSP layers, achieving higher aspect ratios. The presence of a fully cured “top cap (9–10 μm cap labeled by the red arrows)” helps to delay undercut formation in the multilayer stacks.

In this work, we demonstrated two-layer and three-layer PSP. Samples of PSP-13 and PSP-25 were selected since they represent the optimal balance between solved undercut and limited line width expansion cases. As shown in Figure 5, the developed lines of the two-layer PSP-13 (Figure 5a) and PSP-25 (Figure 5b) are still attached to the substrate, with a height of 19.5 ± 0.6 μm and 19.2 ± 0.5 μm, respectively. For the three-layer stack samples, the heights are 30.3 ± 0.5 μm (Figure 5c) and 29.4 ± 0.4 μm (Figure 5d), respectively. The heights are consistent with the expected thickness of ∼10 μm per monolayer of PSP after development.

It is important to note that PSP-0 cannot yield any attached line pattern by the same procedure. The longer development time required for multiple layers causes severe undercut, resulting in the complete peel-off of PSP-0.

Although all of the lines are attached, the cross-section images still show the “etch neck” for all the multiple-layer stacks. This indicates that undercut still exists (see the lower layer width) but is delayed by the completely cured “top cap”, with a similar thickness of 9–10 μm to the monolayer silver oxalate-added PSP in Figure 2. The formation of the thick, etch-free top caps is the main reason for the success of multilayer stack PSP. The thicker top cap can act as a protective, unetchable layer that slows down the etch-neck formation kinetics, preventing the neck width from becoming too narrow. If the neck becomes too narrow, it cannot provide sufficient mechanical support to the upper part of the multilayer structure, leading to structural collapse. Adding silver oxalate has been shown to increase the thickness of the top cap, as indicated by all of the aforementioned results. Therefore, to further increase the aspect ratios of conductive line patterns, new strategies should be explored to enhance the light penetration depth in PSP. In this work, the maximum light penetration depth achieved with the presence of silver oxalate is about 10 μm.

We further annealed the multilayer PSP into metallic silver lines via thermal elimination on all the organics (Figure S1). The resulting heights correspond to the number of layers that are stacked. For example, the height of the two-layer PSP-25 (Figure S1) is 12.2 ± 0.2 μm, approximately two times higher than monolayer PSP-25 (i.e., 7.1 ± 0.4 μm). The bulk resistivity of the single-layer and two-layer stacked PSP-25 is summarized in Table S1. The bulk resistivity decreases as the number of stacking layers of the PSP increases.

## Conclusion

In this research, we have successfully developed a molecular approach to address the persistent undercut issue in the field of PSP. By replacing micrometer-sized silver powders with 25% silver oxalate, we achieved an optimal undercut index of 1.1, nearly reaching the undercut-free index of 1.0. Resolving the undercut issue significantly improved the attachment of line patterns, enhancing both single-line and fringe pattern resolutions. Furthermore, solving the undercut problem made it possible to achieve high aspect ratio lines through multiple-layer stacking of PSP. We also identified a side effect of line width expansion when using silver oxalate to inhibit undercut, likely due to light scattering by the nanosized silver particles. The experimental results suggest that this side effect may be specific-dependent. Therefore, future studies should focus on exploring different silver organic complexes to mitigate the line width expansion issue. Achieving an even higher aspect ratio for the line patterns is limited by the light penetration depth, so future work should focus on developing new methodologies or complexes to overcome this limitation.

Since the undercut of PSP directly correlates with adhesion, we decided not to combine this research with a glass frit study. Yet, we recognize the positive role of using a glass frit in PSP to achieve improved, reasonable adhesion in the future research direction. Additionally, while glass frit can improve the adhesion of the silver line pattern, it is also expected to increase the resistance in the final products.

## Supporting Information Available

Cross-sectional SEM images of PSP-25 after sintering, SEM images of the as-synthesized silver oxalate with the size distribution, UV–vis transmittance of PSP samples, SEM of micron silver powder with size of 1.3 ± 2 μm, comparison of bulk resistivity of single- and two-layer PSP-25 with that of the commercial products (PDF)
