# Supplementary material for: Silver–Organic Complex in Photosensitive Silver Pastes for Enhanced Resolution and Aspect Ratio
Source: Langmuir. 2024 Aug 13;40(34):18254–61. doi: 10.1021/acs.langmuir.4c02158 (PMC11412270; doi:10.1021/acs.langmuir.4c02158)
Supplement: Supplementary file 1 — la4c02158_si_001.pdf [file la4c02158_si_001.pdf]

Supporting Information

Silver-Organic Complex in Photosensitive Silver Pastes for Enhanced Resolution and Aspect Ratio

Jyun-Hao Chen[+], Yen-Ting Liu[+], Chia-Chun Hsieh, Yi-Cheng Chou, and Chun-Hu Chen*

Department of Chemistry, National Sun Yat-sen University, Kaohsiung, Taiwan 80424

*E-mail: chunhu.chen@mail.nsysu.edu.tw

[+]contributed equally

Table of Content

Figure S1. Cross-sectional SEM images of PSP-25 after sintering. (a) Single-layer PSP-25, showing a line thickness of 7.1 ± 0.4 μm after sintering. (b) Two-layer PSP-25, showing a line thickness of 12.2 ± 0.2 μm after sintering. The images demonstrate that the line thickness of two-layer stack approximately doubles as compared to the single layer ones. S-3

Figure S2. SEM images of (a) the as-synthesized silver oxalate showing elongated particle shape with size distribution of (b) the short axis and (c) long axis..............S-4

Figure S3. UV-Vis Transmittance of PSP samples and the list of the transmittance at 405 nm. S-5

Figure S4. The SEM image of micron silver powder (particle size 1.3±0.2 μm) used for PSP preparation without any grinding procedure. S-6

Table S1. Comparison of bulk resistivity of single- and two-layer PSP-25 with that of the commercial products. S-7

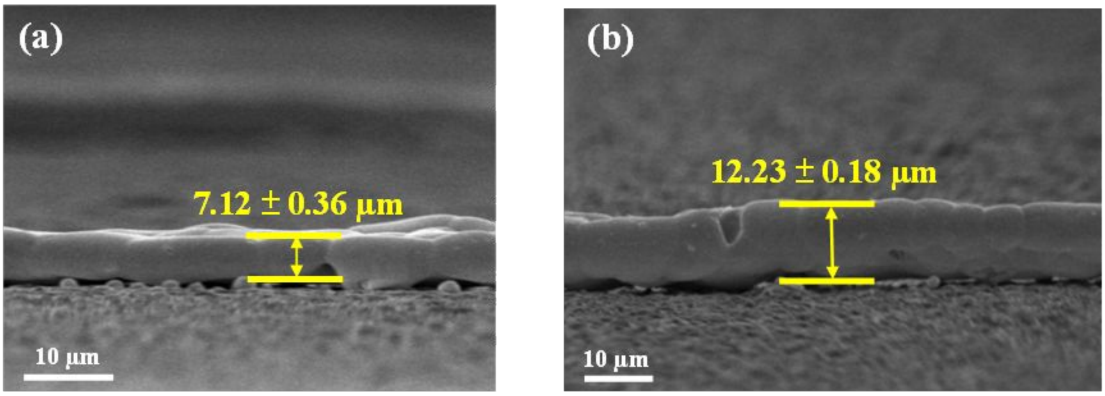

Figure S1. Cross-sectional SEM images of PSP-25 after sintering. (a) Single-layer PSP-25, showing a line thickness of 7.1 ± 0.4 µm after sintering. (b) Two-layer PSP-25, showing a line thickness of 12.2 ± 0.2 µm after sintering. The images demonstrate that the line thickness of two-layer stack approximately doubles as compared to the single layer ones.

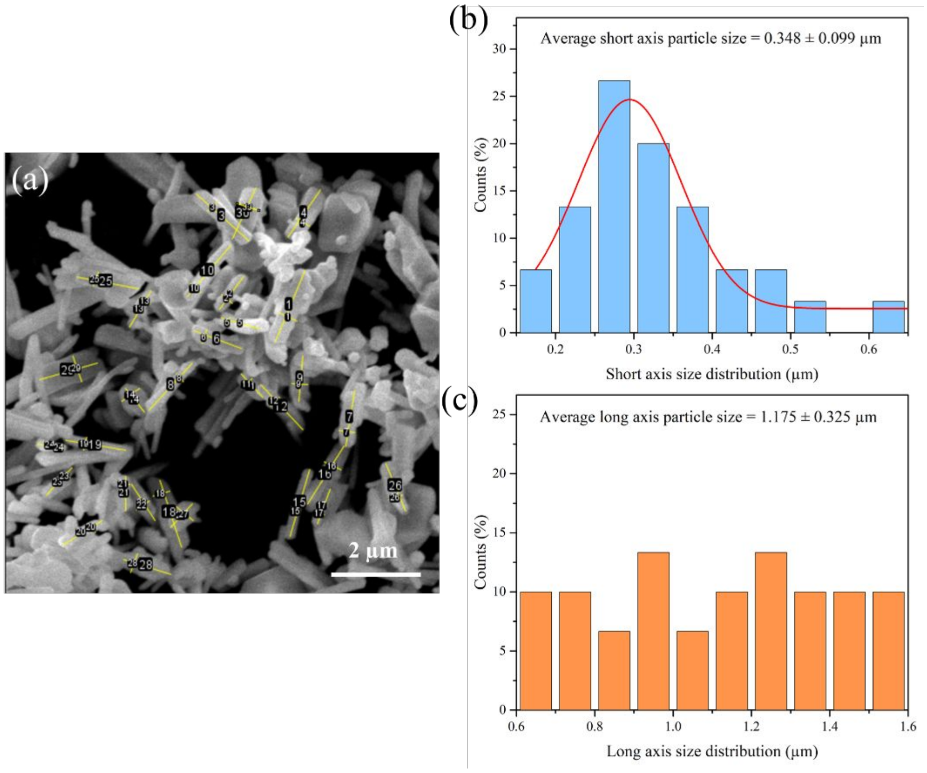

Figure S2. SEM images of (a) the as-synthesized silver oxalate showing elongated particle shape with size distribution of (b) the short axis and (c) long axis.

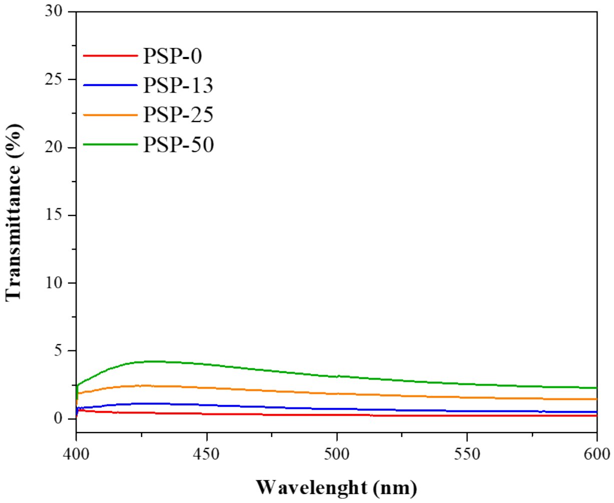

Sample	UV-Vis Transmittance (%) at 405 nm
PSP-0	0.56
PSP-13	0.83
PSP-25	2.03
PSP-50	2.94

Figure S3. UV-Vis Transmittance of PSP samples and the list of the transmittance at 405 nm.

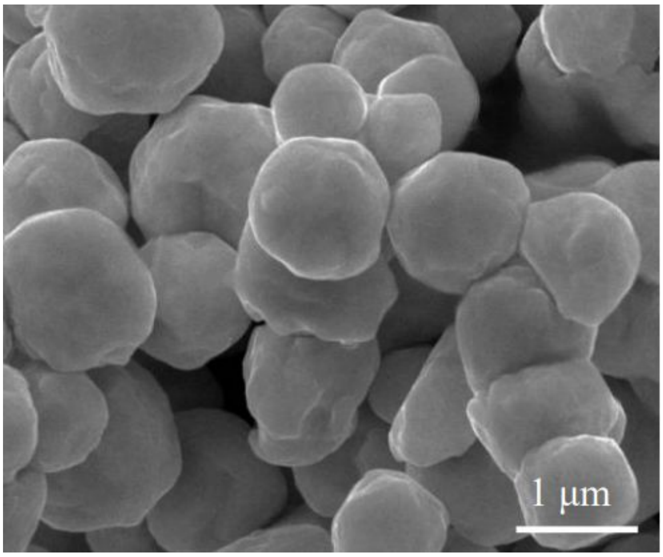

Figure S4. The SEM image of micron silver powder (particle size 1.3±0.2 μm) used for PSP preparation without any grinding procedure.

Table S1. Comparison of bulk resistivity of single- and two-layer PSP-25 with that of the commercial products.

Sample	PSP-25 single-layer	PSP-25 two-layer	Raybrid by Toray [1]
Bulk resistivity (Ω%cm)	$5.51 \pm 0.73 \times 10^{-5}$	$2.26 \pm 0.23 \times 10^{-5}$	$3 – 8 \times 10^{-5}$

Reference:

1. https://www.electronics.toray/en/products/raybrid/rayb_01.html
